# Risk Factors of Orofacial Pain Symptoms Among Older Adults at the End of Life

**DOI:** 10.1093/geroni/igaa057.3048

**Published:** 2020-12-16

**Authors:** Yaolin Pei, Bei Wu

**Affiliations:** New York University, New York, New York, United States

## Abstract

The aims of this study were to examine the prevalence of orofacial pain symptoms in Chinese older adults at the end of life, and to investigate risk factors related to orofacial pain. The sample derived from the Chinese Longitudinal Healthy Longevity Survey (CLHLS), a national respresentative sample of the oldest-old. The results showed that the 6-month prevalence of pain when chewing or biting at the end of life was 11.1%, and the rate was 5% for jaw joint pain/facial pain. Lower SES, smokers, and having chronic diseases were associated with having orofacial symptoms. Unexpectedly, the results revealed that dentate older adults (retain at least one natural tooth) who brushed their teeth more often were more likely to have orofacial symptoms. Older adults have poor oral health, particularly at the end of their life. This study highlights the importance of improving oral health for vulnerable older adults.

